# Low TGF-β1 plasma levels are associated with cognitive decline in Down syndrome

**DOI:** 10.3389/fphar.2024.1379965

**Published:** 2024-03-21

**Authors:** Margherita Grasso, Annamaria Fidilio, Francesca L’Episcopo, Marilena Recupero, Concetta Barone, Maria Giulia Bacalini, Cristina Benatti, Maria Concetta Giambirtone, Giuseppe Caruso, Donatella Greco, Santo Di Nuovo, Corrado Romano, Raffaele Ferri, Serafino Buono, A. Claudio Cuello, Johanna M. C. Blom, Fabio Tascedda, Pier Vincenzo Piazza, Rafael De La Torre, Filippo Caraci

**Affiliations:** ^1^ Oasi Research Institute-IRCCS, Troina, Italy; ^2^ IRCCS Istituto Delle Scienze Neurologiche di Bologna, Bologna, Italy; ^3^ Department of Biomedical, Metabolic and Neural Sciences, University of Modena and Reggio Emilia, Modena, Italy; ^4^ Department of Drug and Health Sciences, University of Catania, Catania, Italy; ^5^ Department of Educational Sciences, University of Catania, Catania, Italy; ^6^ Department of Biomedical and Biotechnological Sciences, University of Catania, Catania, Italy; ^7^ Department of Pharmacology, McGill University, Montreal, QC, Canada; ^8^ Department of Life Sciences and Center for Neuroscience and Neurotechnology, University of Modena and Reggio Emilia, Modena, Italy; ^9^ Aelis Farma, Bordeaux, France; ^10^ Integrative Pharmacology and Systems Neurosciences Research Group, Neurosciences Research Program, Hospital del Mar Research Institute /HMRI, Barcelona, Spain

**Keywords:** Down’s syndrome, cognitive decline, TGF-β1, PBMCs, blood-based biomarker

## Abstract

Almost all individuals with Down’s syndrome (DS) show the characteristic neuropathological features of Alzheimer’s disease (AD) by the age of 40, yet not every individual with DS experiences symptoms of AD later in life. Similar to neurotypical developing subjects, AD in people with DS lasts for a long preclinical phase in which biomarkers follow a predictable order of changes. Hence, a prolonged asymptomatic period precedes the onset of dementia, underscoring the importance of identifying new biomarkers for the early detection and monitoring of cognitive decline in individuals with DS. Blood-based biomarkers may offer an alternative non-invasive strategy for the detection of peripheral biological alterations paralleling nervous system pathology in an early phase of the AD continuum. In the last few years, a strong neurobiological link has been demonstrated between the deficit of transforming growth factor-β1 (TGF-β1) levels, an anti-inflammatory cytokine endowed with neuroprotective activity, and early pro-inflammatory processes in the AD brain. In this clinical prospective observational study, we found significant lower plasma TGF-β1 concentrations at the first neuropsychological evaluation (baseline = T0) both in young adult DS individuals (19–35 years) and older DS subjects without AD (35–60 years) compared to age- and sex-matched healthy controls. Interestingly, we found that the lower TGF-β1 plasma concentrations at T0 were strongly correlated with the following cognitive decline at 12 months. In addition, in young individuals with DS, we found, for the first time, a negative correlation between low TGF-β1 concentrations and high TNF-α plasma concentrations, a pro-inflammatory cytokine that is known to be associated with cognitive impairment in DS individuals with AD. Finally, adopting an *ex vivo* approach, we found that TGF-β1 concentrations were reduced in parallel both in the plasma and in the peripheral blood mononuclear cells (PBMCs) of DS subjects, and interestingly, therapeutic concentrations of fluoxetine (FLX) applied to cultured PBMCs (1 µM for 24 h) were able to rescue TGF-β1 concentrations in the culture media from DS PBMCs, suggesting that FLX, a selective serotonin reuptake inhibitor (SSRI) endowed with neuroprotective activity, might rescue TGF-β1 concentrations in DS subjects at higher risk to develop cognitive decline.

## 1 Introduction

Down syndrome (DS) is the most common genetic cause of intellectual disability attributable to a partial or complete trisomy of chromosome 21 that affects approximately one in 700 individuals at birth (Parizot et al., 2019). The majority of individuals with DS typically exhibit moderate-to-severe intellectual functioning, with an intellectual quotient (IQ) generally falling within the range of 30–70 (Grieco et al., 2015). Intellectual disability also includes deficits in associative and working memory, executive function, and episodic and explicit memories (Contestabile et al., 2010; Colizzi et al., 2020). Furthermore, this is exacerbated by the fact that a significant number of individuals with DS will experience the onset of Alzheimer’s disease (AD) in their early adulthood (Caraci et al., 2017). By the age of 40, nearly all DS subjects display the characteristic neuropathological features of AD, such as the deposition of senile plaques containing amyloid-beta (Aβ) peptide, chronic oxidative stress, and neurofibrillary tangle formation (Franceschi et al., 2019; Lott and Head, 2019). Previous studies suggested that not all individuals with DS experience cognitive decline associated with AD during adulthood (Iulita et al., 2016a), whereas more recent studies found that all individuals with DS will develop AD in their adulthood (average 53.8 years) and also that this is independent that there is an asymptomatic period like for neurotypical developing people, although AD progression is much faster (Iulita et al., 2022). The increased incidence of early-onset AD in individuals with DS is attributed to the overexpression of amyloid precursor protein (APP), which is encoded by the APP gene on chromosome 21, leading to elevated concentrations and deposition of the cerebral Aβ peptide (Caraci et al., 2017). Given the high-risk nature of this population, it is likely that a protracted asymptomatic phase precedes the onset of dementia (Karmiloff-Smith et al., 2016; Fortea et al., 2020). The diagnosis of AD in individuals with DS is likely complicated primarily by the pre-existing intellectual disability and cognitive decline associated with aging (Zigman, 2013).

In this scenario, the identification of biomarkers for cognitive decline prediction is essential among aging individuals with DS. Promising results have been achieved with brain imaging and cerebrospinal fluid (CSF) biomarkers, demonstrating good diagnostic performance in detecting AD in adults with DS (Lee et al., 2017; Fortea et al., 2018). However, blood-based biomarkers are advantageous due to their accessibility, lower cost, and better tolerance compared to neuroimaging and CSF approaches. Blood-based biomarkers have been proposed as non-invasive screening tools to detect early cognitive deficits and prodromal AD-type dementia in adult subjects with DS (Petersen and O'Bryant, 2019). Plasma neurofilament light chain (NfL) concentrations have shown an excellent diagnostic and prognostic performance for symptomatic AD in DS and have been proposed as plasma biomarkers (Carmona-Iragui et al., 2021).

Cognitive deficits and AD-related cognitive decline in DS individuals are associated with neuroinflammation (Caraci et al., 2017), a deficit of neurotrophic factors (Hartley et al., 2015; Iulita et al., 2016a) and an “inflammatory endophenotype” (Ahmed et al., 2021) with increased concentrations of pro-inflammatory cytokines (e.g., IL-1 and TNF-α) (Iulita et al., 2016b). However, no studies have been conducted to understand whether a deficit of anti-inflammatory cytokines can contribute to cognitive deficits in DS.

Transforming growth factor-β1 (TGF-β1) is an anti-inflammatory cytokine that exerts neuroprotective effects against amyloid-induced neurodegeneration and plays a key role in memory formation and synaptic plasticity (Caraci et al., 2012; Caraci et al., 2018). The impairment of the TGF-β1 pathway is an early occurrence linked to neuroinflammation and cognitive decline in AD brains (Caraci et al., 2012; Caraci et al., 2018; Kapoor and Chinnathambi, 2023). However, it remains unclear whether a deficiency in TGF-β1 contributes to the onset or progression of cognitive impairment in DS subjects.

In addition to plasma, some blood cells like peripheral blood mononuclear cells (PBMCs) can be suggested as a straightforward *ex vivo* model for discovering new pharmacological targets and biomarkers of early cognitive decline in individuals with DS. This is because PBMCs may mirror the biological changes observed at the central nervous system (CNS) level (Rodrigues et al., 2014; Esteras et al., 2016).

Based on these observations, in the present paper, we examined both TGF-β1 and TNF-α plasma concentrations in young adults (19–35 years) and older adults (35–60 years) with DS in comparison to age- and sex-matched healthy controls (HCs). Additionally, we studied the correlation between low TGF-β1 concentrations and elevated TNF-α plasma concentrations specifically in young adult DS individuals and the correlation between these cytokines and cognitive decline. We also tested for the first time *ex vivo* whether therapeutic concentrations of FLX, a selective serotonin reuptake inhibitor (SSRI) known to elevate TGF-β1 in rodents (Torrisi et al., 2019), could rescue TGF-β1 concentrations in the culture medium of PBMCs from individuals with DS.

## 2 Materials and methods

### 2.1 Study cases

Young (aged 19–35 years) and older adults (aged 35–60 years) with DS were recruited by the Oasi Research Institute-IRCCS, Associazione Oasi Maria SS, Troina, Sicily, Italy, totaling 49 cases who assented to blood extraction. Participants or their legal representative gave written informed consent, as approved by the Ethics Committee of the Oasi Research Institute-IRCCS (2021/02/16/CE-IRCCS-OASI/PA17, grant no.: GR-2019-12369983). The control groups consist of healthy volunteers age- and sex-matched (age range 19–60 years, n = 44) with neither karyotype abnormalities nor neurological deficits.

The Italian version of the Leiter International Scale-Third Edition was used for the estimation of the IQ in subjects with DS. This scale measures cognitive abilities, non-verbal intelligence, non-verbal memory, and attention (Cornoldi et al., 2016). The Test for Severe Impairment (TSI) evaluates cognitive function in patients with severe cognitive impairment, particularly in advanced dementia. It was administered to assess global cognitive function and cognitive decline both in young and older adult subjects with DS. Following neurological examination, DS individuals with AD-related cognitive decline (35–60 years) were categorized in early-onset, middle-stage, and late-stage dementia, according to the Dementia Scale for Down syndrome (DSDS). TSI and DSDS were administered to assess the rate of cognitive decline in this population.

The TSI evaluation in subjects with DS was performed at the time of plasma collection (baseline, T0) and at the follow-up period (12 months from T0). The Italian validated version of the TSI (range 0–24) was used to assess the cognitive decline in all DS individuals, and lower scores indicated a greater cognitive impairment (Appollonio et al., 2001).

Participant subjects with DS were classified in young adults (19–35 years, n = 26) and older adults without (35–60 years, n = 14) or with AD-related cognitive decline DS-AD (35–60 years, n = 9). In addition, the rate of cognitive decline was calculated as delta (d) TSI by subtracting the cognitive score obtained during the neuropsychological evaluation at the baseline period (T0) from those at the follow-up interval (T1) and dividing it by the months (in our cohort = 12 months) from T0, as previously described (Iulita et al., 2016b). In total, 36 individuals with DS [young adults n = 20 and older adults without (n = 10) or with AD-related cognitive decline (n = 6)] out of 49 subjects recruited at T0 completed the neuropsychological evaluation at 12 months (dropouts were mainly due to the COVID-19 pandemic).

Table 1 shows demographic and clinical characteristics of individuals with DS included in this study.

**TABLE 1:** Demographic and clinical information on DS participants. Number of cases, age (±SEM) at baseline (T0), gender, I.Q. T0 (mean), and TSI T0 (mean ± SEM) of HC (19–35 years), young adult individuals with DS (DS (19–35 years)), HC (35–60 years), older adult individuals DS without AD-related cognitive decline (DS (35–60 years)), and older adult individuals with DS and AD-related cognitive decline (DS-AD (35–60 years)). N/A: not applicable. Ordinary one-way ANOVA, Bonferroni’s *post hoc*, *p < 0.05 older adult DS-AD (35–60 years) vs. older adult DS (35–60 years), and #*p* < 0.01 older adult DS-AD (35–60 years) vs. young adult DS (19–35 years).

	HC (19–35 years)	Young adult DS (19–35 years)	HC (35–60 years)	Older adult DS (35–60 years)	Older adult DS-AD (35–60 years)
**Number of cases**	22	26	22	14	9
**Age (±SEM) T0**	27.9 ± 0.9	28.5 ± 0.8	45.5 ± 1.7	43.9 ± 1.7	45.9 ± 3.2
**Gender**	12 M–10 F	13 M–13 F	10 M–12 F	8 M–6 F	4 M–5 F
**I.Q. T0 (mean)**	N/A	44	N/A	42	41
**TSI T0 (mean ± SEM)**	N/A	20.3 ± 0.8	N/A	17.7 ± 1.3	11.1 ± 3.1 *#

### 2.2 Plasma sample collection and PBMCs isolation

Plasma samples used to perform enzyme-linked immunosorbent (ELISA) assay were collected according to standard procedures. In brief, fasting venous blood was collected in lavender EDTA-K2 BD Vacutainer tubes and centrifuged at 1,900 rpm for 10 min for the separation of the plasma component from the cellular constituents. After second centrifugation (3,900 rpm for 10 min) to purify from biological debris, plasma samples were separated in aliquots and stored at −80°C until use. The blood was used to isolate peripheral blood mononuclear cells (PBMCs) using the Lympholyte®-H density gradient separation medium (Cedarlane, Burlington, NC, United States, cod. CL5016), according to the manufacturer’s instructions, with slight modifications. In brief, the blood was diluted with an equal volume of 1× PBS, mixed gently, and added to one part of Lympholyte®-H. After the centrifugation step (400 rcf for 30 min), a well-defined lymphocyte layer appeared at the interface, which was removed and transferred using a serological pipette to a new sterile centrifuge tube. After washing using 5 mL of 1× Red Blood Cell Lysis Buffer (Abcam, ab204733) for optimal lysis of erythrocytes in a single-cell suspension and after two additional washing steps (1× sterile PBS, 1,600 rpm for 10 min), the isolated PBMC fraction was counted using the LUNA-II Automated Cell Counter (Logos Biosystems).

### 2.3 Drug treatment of PBMC cultures

PBMCs obtained from DS and HC subjects were maintained in fresh culture medium (RPMI 1640 medium (Merck KGaA, cod. R8758) supplemented with 10% fetal bovine serum (FBS, Gibco, Thermo Fisher Scientific, cod. A5256801), 1× penicillin/streptomycin (Merck KGaA, cod. 4333), 1× L-glutamine (BioConcept, cod. 5-10K00-H), and 1× amphotericin b (BioConcept, cod. 4-05F00-H)) for 1 week and then plated at a density of 1×10^5^/dish. After 24 h of incubation at 37°C, PBMC cultures were treated with FLX 1 µM. Fluoxetine hydrochloride (Merck KGaA, cod. F132) was dissolved in DMSO at the final concentration of 0.1% (Caraci et al., 2016) and applied to the PBMC cultures at the final concentration of 1 µM. The therapeutic concentration and a 24-h treatment period were chosen based on our previous *in vitro* studies conducted in neuronal cultures where we assessed the dose response of this antidepressant drug, showing a maximal neuroprotective activity at 1 µM (Caraci et al., 2016). Conditioned-culture media were collected before (baseline, T0) and after FLX treatment (24 h, T1) for ELISA analysis.

### 2.4 Analysis of TGF-β1 and TNF-α concentrations

The quantitative determination of active human TGF-β1 in the plasma obtained from individuals with DS and HC at baseline (T0 = baseline) was carried out using the ELISA kit (Bio-Techne, R&D system, cod. DB100 B), according to manufacturer’s instructions. TGF-β1 concentrations were measured both in diluted plasma samples (1:10) and in PBMC cultured media. The optical density of each well was determined using a microplate reader Synergy HT (Agilent BioTek, Santa Clara, CA, United States) set to 450 nm, 540, and 570 nm.

The plasma concentrations of the pro-inflammatory cytokine TNF-α at baseline (T0 = baseline) were determined using the human TNF-α ELISA kit (Bio-Techne, R&D system, cod. DTA00D), following the protocol detailed by the manufacturer.

### 2.5 Statistical analysis

Data obtained from ELISA assays in plasma samples and in PBMC cultured media were analyzed using one-way analysis of variance (ANOVA), followed by the non-parametric multiple comparisons (Kruskal–Wallis test) using Dunn’s *post hoc* correction test. Moreover, to examine TGF-β1 and TNF-α plasmatic concentrations in young adults with DS (19–35 years), we used the non-parametric Mann–Whitney unpaired *t*-test. Ordinary one-way ANOVA followed by Bonferroni’s multiple-comparison test was used to analyze demographic and clinical characteristics of DS participants. The Spearman rank test and Pearson’s correlation were used for bivariate correlation analyses. GraphPad Prism software^®^ 9.0 (GraphPad, La Jolla, CA, United States) was used to perform the analyses. Results from ELISA assay are reported as means ± S.E.M, and only *p* values <0.05 were considered statistically significant. Outliers were removed by applying the interquartile range (IQR) method.

## 3 Results

Individuals with DS and a diagnosis of AD (35–60 years) exhibited the lowest TSI scores at T0 compared to individuals with DS of the same age range, but without AD-related cognitive decline (*p* < 0.05), and to young adults with DS (19–35 years) (*p* < 0.01), indicative of moderate cognitive impairment in individuals with DS-AD (Table 1 contains a summary of the demographic and clinical characteristics of DS participants). At the time of the initial plasma collection (T0), young adults (19–35 years) and older adults with DS (35–60 years, with or without AD-related cognitive decline) showed a similar IQ. A significant difference was detected between older and young individuals with DS in the mean of TSI scores at T0 (*p* < 0.05, Table 2). Moreover, we found an inverse correlation between age and TSI score at T0 (Pearson r −0.41, *p* < 0.01).

**TABLE 2:** Intelligence quotient (Intellectual quotient) and TSI score at T0 in young and older individuals with DS. Mean (±SEM) of IQ and TSI score at the baseline neurophysiological evaluation in young adult individuals with DS (19–35 years) (DS YOUNG (19–35 years)) and older adult individuals with DS (35–60 years) with or without AD-related cognitive decline (DS OLDER (35–60 years)). Ordinary one-way ANOVA, Bonferroni’s *post hoc*, *p < 0.05 DS OLDER (35–60 years) vs. DS young (19–35 years).

	I.Q. TOT	I.Q. DS young (19–35 years)	I.Q. DS older (35–60 years)
**(Mean ± SEM)**	42.9 (±0.5)	43.7 (±0.7)	41.7 (±0.4)

	TSI TOT (T0)	TSI DS young (19–35 years**)**	TSI DS older (35–60 years**)**
**(Mean ± SEM)**	17.9 (±0.9)	20.3 (±0.8)	15.1 (±1.5) *

### 3.1 Plasma TGF-β1 concentrations are lower in adult DS individuals

**FIGURE 1:**
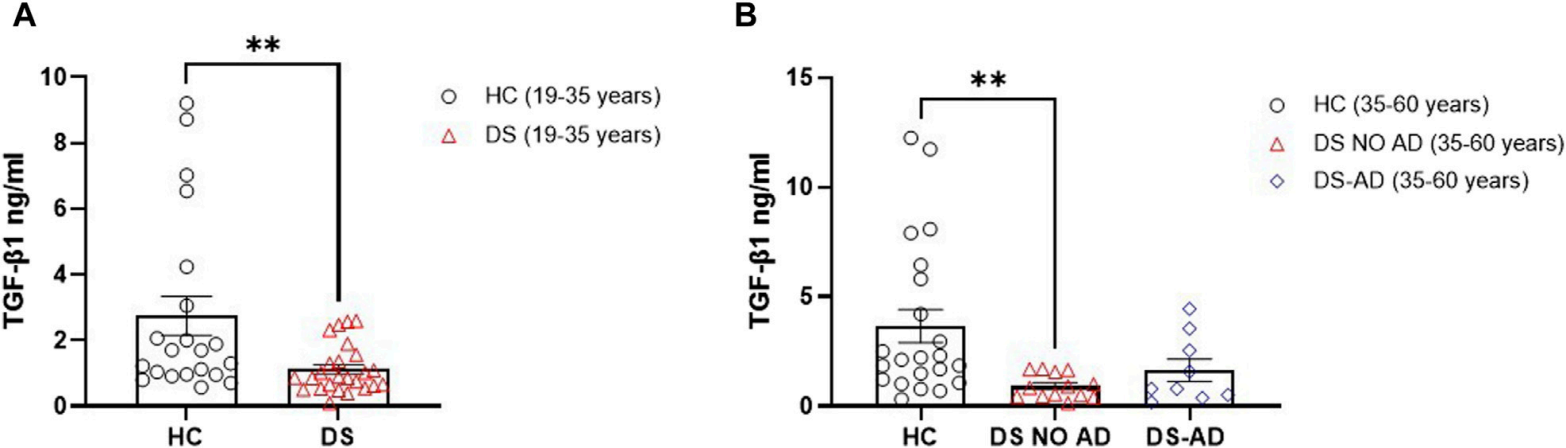
Deficit of TGF-β1 concentrations in the plasma of individuals with DS. Plasma TGF-β1 concentrations measured by ELISA assay in **(A)** young adult individuals with DS (19–35 years, n = 25) and healthy controls (HC) (19–35 years, n = 21); *t*-test unpaired, non-parametric test, Mann–Whitney test, **p < 0.01 DS (19–35 years) vs. HC; **(B)** older adult with or withour Alzheimer’s disease (AD)-related cognitive decline individuals with DS (35–60 years, n = 9 and n = 13, respectively) and HC (35–60 years, n = 22); one-way ANOVA, non-parametric test, Kruskal–Wallis test, Dunn’s *post hoc* test, **p < 0.01 DS NO AD (35–60 years) vs. HC (35–60 years), not significant (n.s.) DS-AD (35–60 years) vs. HC (35–60 years). All data are shown as mean ± SEM.

In order to substantiate the hypothesis suggesting differences of TGF-β1 plasma concentrations in the early preclinical stages of cognitive decline in individuals with DS, we assessed this anti-inflammatory cytokine in individuals with DS, distinguishing those with or without AD-related cognitive decline. Intriguingly, a statistically significant reduction is observed in TGF-β1 plasma concentrations at baseline (baseline = T0) in both young adults (19–35 years) (Figure 1A; *p* < 0.01) and older adults (35–60 years) with DS without AD-related cognitive decline (Figure 1B; *p* < 0.01), as compared to age- and sex-matched HCs. However, this decrease in TGF-β1 plasma concentrations did not reach statistical significance in older individuals with DS experiencing AD-related cognitive decline (DS-AD) compared to HCs.

**FIGURE 2:**
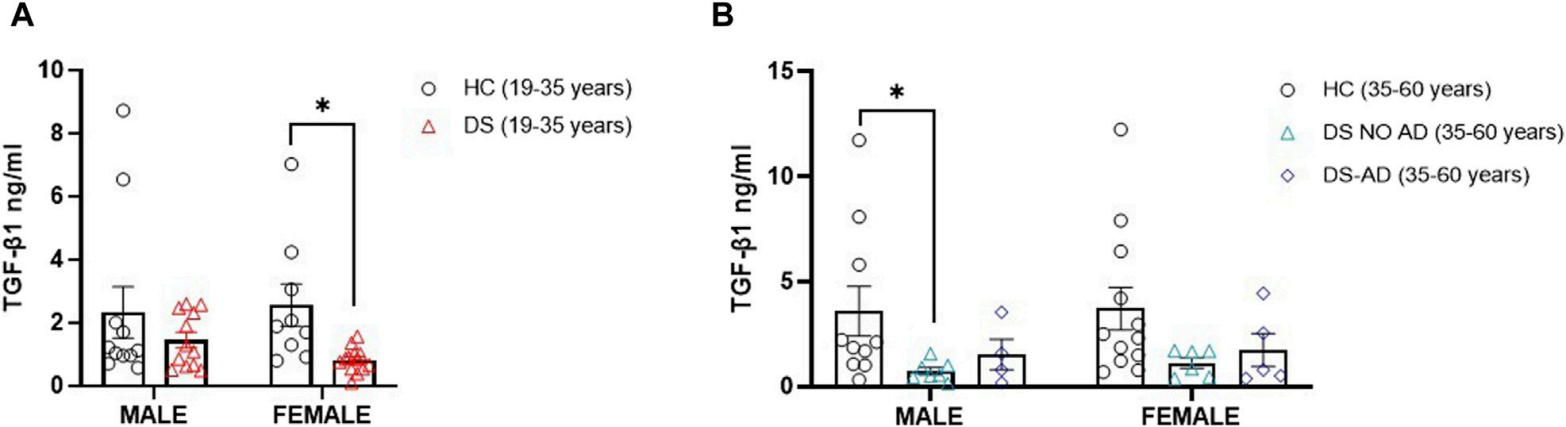
Plasma TGF-β1 concentrations at baseline following gender. Plasma TGF-β1 concentrations measured by ELISA assay in **(A)** male and female young adults with DS (19–35 years, male n = 12, female n = 13), *p < 0.05 female DS (19–35 years) vs. female healthy controls (HCs) (19–35 years, n = 9), n.s. male DS (19–35 years) vs. male HC (19–35 years, n = 11); and in **(B)** male and female older adult individuals with DS (35–60 years, male n = 7, female n = 6), older adult DS-AD (35–60 years, male n = 4, female n = 5) and HC (35–60 years, male n = 10, female n = 12), *p < 0.05 male DS NO AD (35–60 years) vs. HC male (35–60 years), n.s. male DS-AD (35–60 years) vs. male HC (35–60 years), and n.s. in the female group. One-way ANOVA, non-parametric test, Kruskal–Wallis test, and Dunn’s *post hoc* test are conducted. All data are shown as mean ± SEM.

Then, we explored the potential association between gender and the lower TGF-β1 plasma concentrations at baseline. Strikingly, we observed a notable reduction in TGF-β1 concentrations among young adult female subjects with DS (19–35 years) compared to age- and sex-matched healthy controls (Figure 2A, *p* < 0.05). However, we observed the decrease in TGF-β1 in young adult male subjects with DS relative to their sex-matched control group that was not statistically significant (Figure 2A). Similarly, comparable lower TGF-β1 plasma concentrations were evident solely in older adult male subjects with DS (35–60 years) without AD-related cognitive decline (Figure 2B, *p* < 0.05), while the observed decrease in TGF-β1 concentrations in young adult female subjects (19–35 years) persists in older adult female subjects without AD-related cognitive decline (35–60 years), although this difference did not reach a statistical significance. These findings suggest that plasma TGF-β1 concentrations may be a biomarker in an early phase of cognitive decline, preceding the clinical manifestation of an AD-related phenotype in DS subjects.

### 3.2 Lower TGF-β1 plasma concentrations are associated with an increased rate of cognitive decline in DS

**FIGURE 3:**
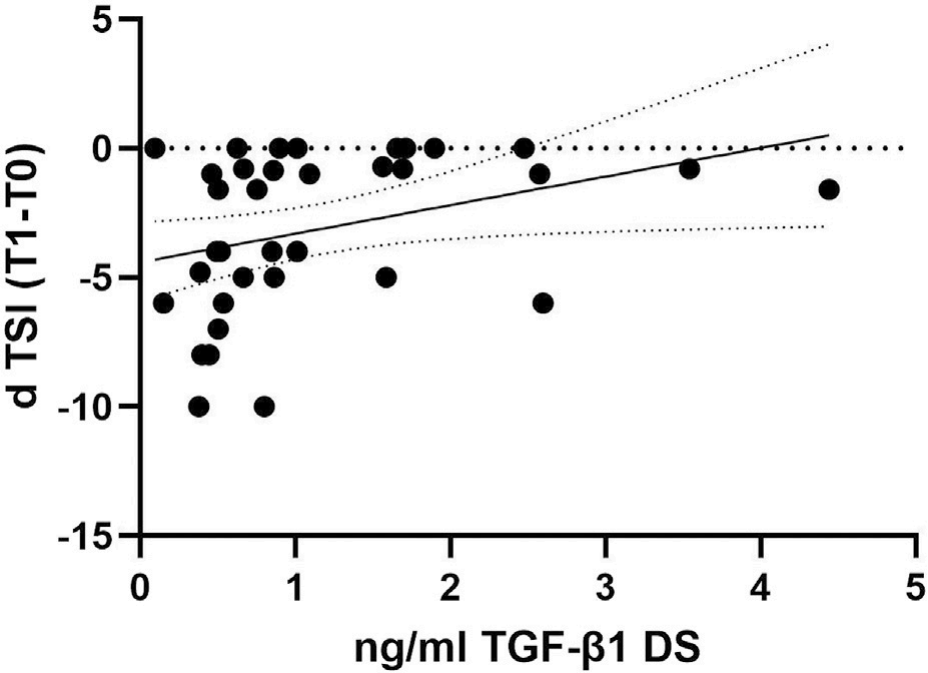
TGF-β1 baseline plasma concentrations correlated with the rate of cognitive decline at the follow-up period. Correlation between the plasmatic concentration of TGF-β1 at baseline (T0) and the rate of cognitive decline calculated for each individual as dTSI (T1 TSI–T0 TSI/12 months) in individuals with DS (n = 36). Simple linear regression, Spearman r 0.4427, 95% CI: 0.07275 to 2.137, **p < 0.01.

To investigate whether the decline in TGF-β1 plasma concentrations can predict cognitive decline progression in DS individuals, we examined the association between plasma concentrations of TGF-β1 at baseline (T0) and the following rate of cognitive decline calculated for each individual as dTSI (DS subjects n = 36). No association was found between age and TGF-β1 concentrations at T0 (Pearson r 0.1124, p = 0.51, DS subjects n = 36). Interestingly, we found that lower TGF-β1 plasma concentrations at T0 were strongly correlated with the cognitive decline assessed by the TSI score at T1 (12 months) in individuals with DS (Figure 3, Spearman r 0.4427, *p* < 0.01), suggesting that a decline in TGF-β1 plasma concentrations could be associated with cognitive decline in adult individuals with DS.

### 3.3 TNF-α plasma concentrations are increased in young adult DS individuals and are inversely related to TGF-β1 concentrations

To evaluate the contribution of neuroinflammatory processes in cognitive decline in individuals with DS, we measured TNF-α plasma concentrations, a well-known pro-inflammatory mediator involved in AD-related cognitive decline in DS (Iulita et al., 2016b).

**FIGURE 4:**
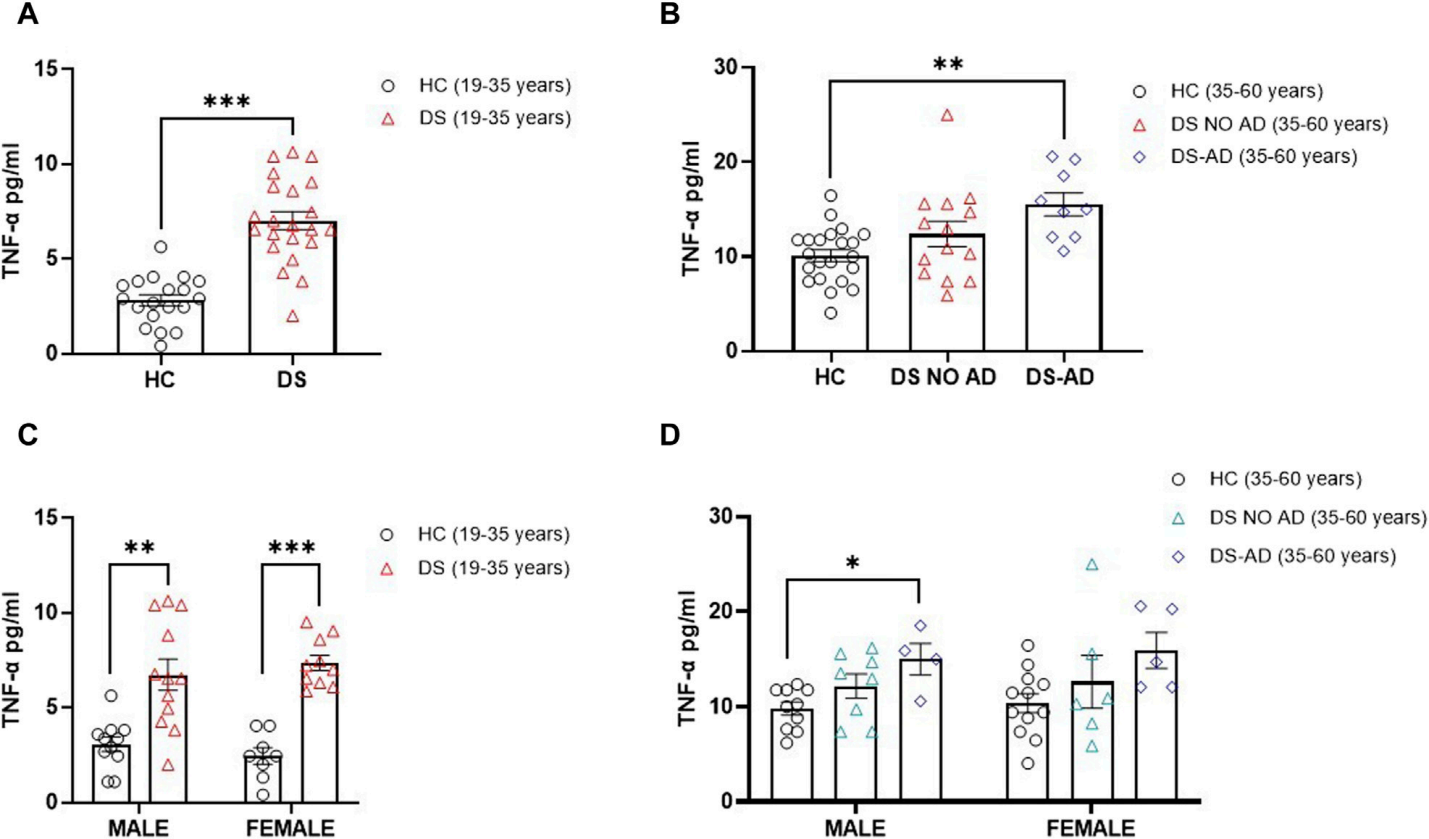
Plasma concentrations of TNF-α in individuals with DS. Plasma TNF-α concentrations measured by ELISA in **(A)** young adult individuals with DS (19–35 years, n = 22) and healthy controls (HC) (19–35 years, n = 19); *t*-test unpaired, non-parametric test, Mann–Whitney test, ***p < 0.001 DS (19–35 years) vs. HC; **(B)** older adult with or withour Alzheimer’s disease (AD)-related cognitive decline in individuals with DS (35–60 years, n = 9 and n = 14, respectively) and HC (35–60 years, n = 22); one-way ANOVA, non-parametric test, Kruskal–Wallis test, Dunn’s *post hoc*, **p < 0.01 DS-AD (35–60 years) vs. HC, n. s. DS NO AD (35–60 years) vs. HC; **(C)** male and female young adult individuals with DS (19–35 years, male n = 12, female n = 10), one-way ANOVA, non-parametric test, Kruskal–Wallis test, Dunn’s *post hoc* test, **p < 0.01 male DS (19–35 years) vs. male HC (19–35 years, n = 11), ***p < 0.001 female DS (19–35 years) vs. female HC (19–35 years, n = 8); **(D)** male and female older adult individuals with DS (35–60 years, male n = 8, female n = 6), and older adult DS-AD (35–60 years, male n = 4, female n = 5); one-way ANOVA, Non-parametric test, Kruskal–Wallis test, Dunn’s *post hoc*, *p < 0.05 male DS-AD (35–60 years) vs. male HC (35–60 years, n = 10), n.s. DS NO AD (35–60 years) vs. male HC, n.s. in the female group compared to female HC (35–60 years, n = 12). All data are shown as mean ± SEM.

In alignment with expectations, our investigation revealed elevated TNF-α concentrations in older adult individuals with DS and AD-related cognitive decline (Figure 4B, *p* < 0.01). Interestingly, an increase in the TNF-α plasma concentration was also observed in young adult individuals with DS (19–35 years) compared to their matched HCs (Figure 4A, *p* < 0.001). Notably, no gender-related differences were discerned in young adult individuals with DS, with both male and female individuals displaying a roughly four-fold increase in TNF-α plasma concentrations compared to age- and sex-matched HCs (Figure 4C, *p* < 0.01 for male individuals, *p* < 0.001 for female individuals). A similar pattern was noted in older adult DS-AD male subjects, whereas no significant increase was observed in older adult DS-AD female subjects (Figure 4D, *p* < 0.05 for male individuals). However, no correlation was observed between the TNF-α plasma concentration at baseline (T0) and the following rate of cognitive decline evaluated as dTSI (Spearman r -0.1053, *p* = 0.5).

**FIGURE 5:**
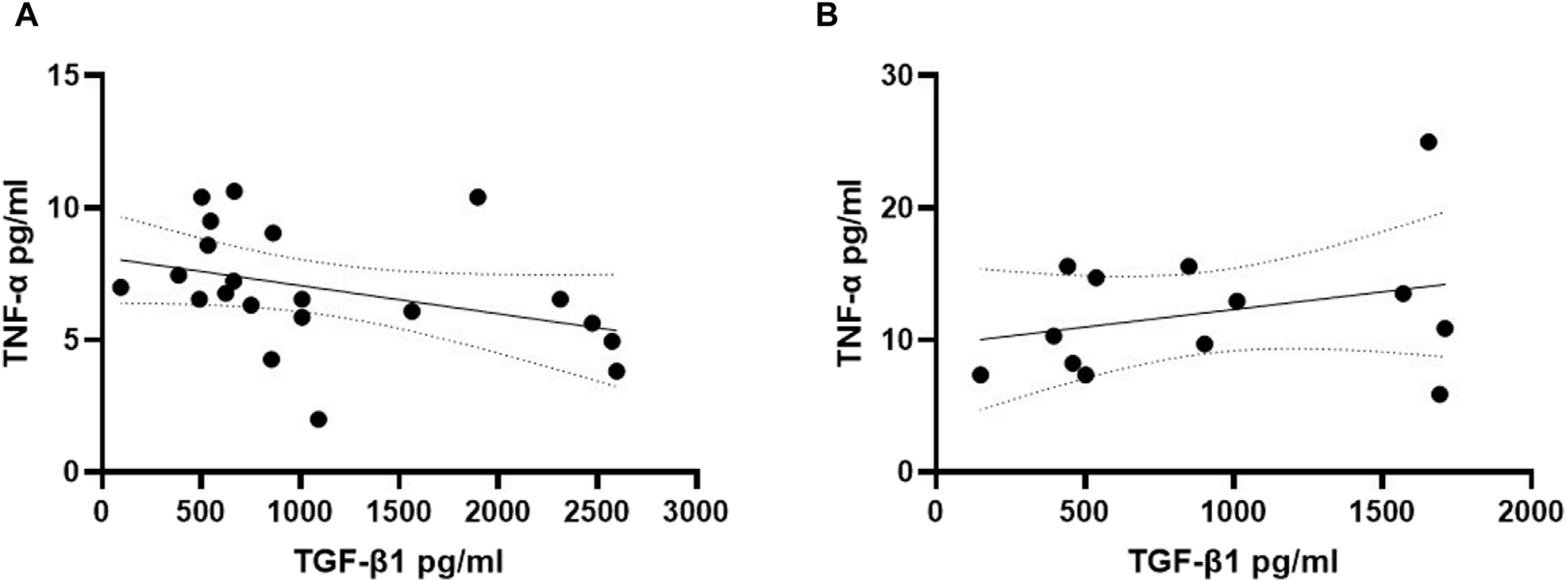
TGF-β1 plasma concentrations correlated with TNF-α plasma concentrations in young adult DS individuals. Correlation between the plasmatic concentration of TGF-β1 and TNF-α at T0 in **(A)** young adult DS individuals (19–35 years, n = 21); simple linear regression, Spearman r −0.5576, 95% CI: −0.8023 to −0.1525, **p < 0.01; and in **(B)** older adult DS (35–60 years, n = 13); simple linear regression, Spearman r 0.1598, 95% CI: −0.4438 to 0.6636, *p* = 0.59.

To explore potential interactions between TGF-β1 and TNF-α in the early phase of cognitive decline in individuals with DS, we investigated the correlation between plasma concentrations of these two cytokines in both young and older adult DS individuals without AD. Interestingly, a negative correlation between low TGF-β1 concentrations and high TNF-α plasma concentrations was observed exclusively in the group of young adult individuals with DS (19–35 years) (Figure 5A, Spearman r= -0.5576, *p* < 0.01), while no such correlation was found neither in older adult individuals with DS without AD-related cognitive decline (Figure 5B, Spearman r 0.1598, *p* = 0.59) nor in older adult DS-AD (35–50 years) (Spearman r = 0.57, *p* = 0.10).

**FIGURE 6:**
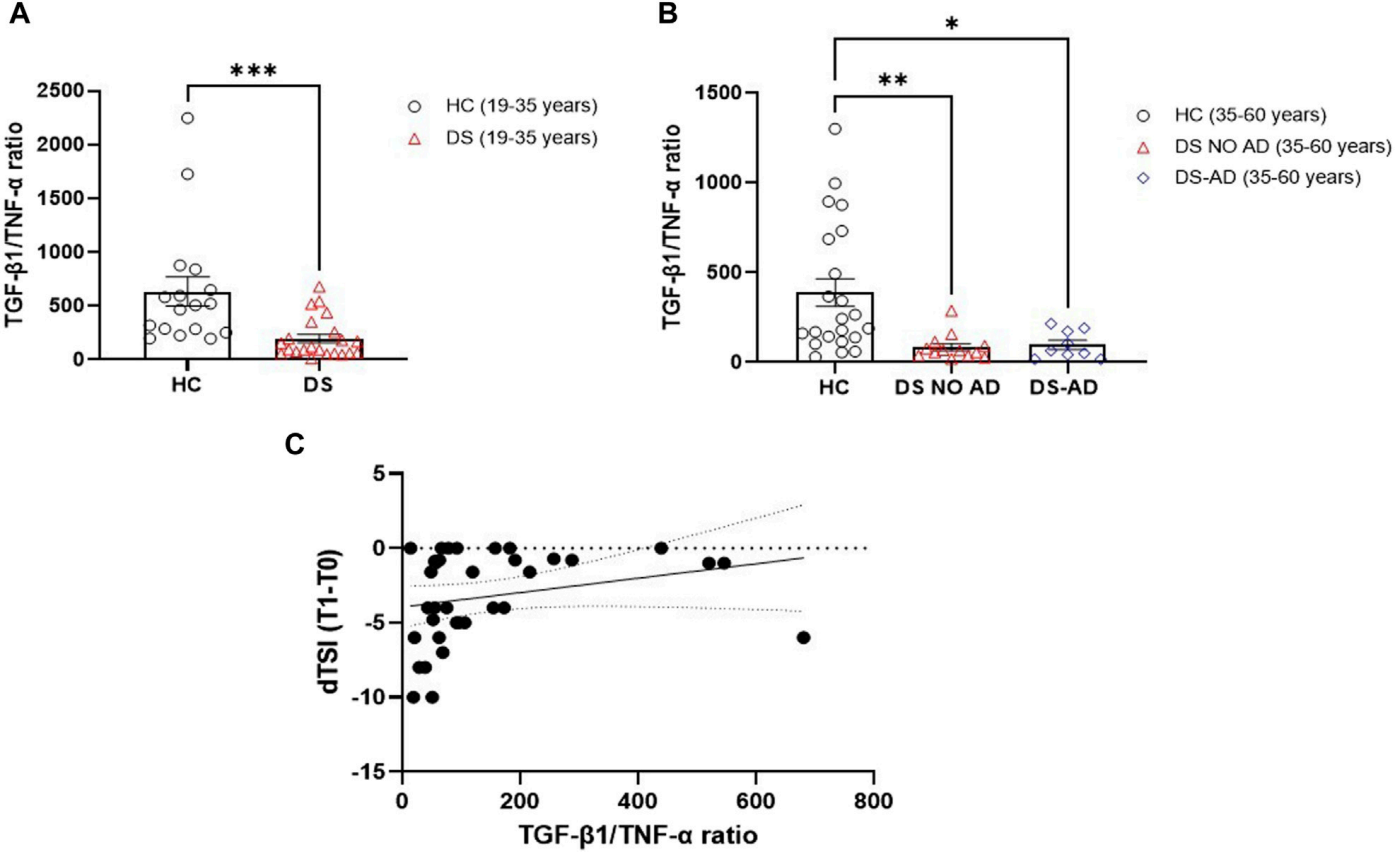
Plasma TGF-β1/TNF-α ratio in the DS population. TGF-β1/TNF-α ratio in **(A)** young adult DS individuals (19–35 years, n = 22), *t*-test unpaired, non-parametric test, Mann–Whitney test, ***p < 0.001 DS (19–35 years) vs. HC (19–35 years, n = 17); and in **(B)** older adult with or withour Alzheimer’s disease (AD)-related cognitive decline DS individuals (35–60 years, n = 9 and n = 13, respectively), one-way ANOVA, non-parametric test, Kruskal–Wallis test, Dunn’s *post hoc*, **p < 0.01 DS NO AD (35–60 years) vs. HC (35–60 years, n = 22), *p<0.05 DS-AD (35–60 years) vs. HC (35–60 years). **(C)** Correlation between TGF-β1/TNF-α ratio and the rate of cognitive decline calculated for each individual as dTSI (T1 TSI–T0 TSI/12 months) in individuals with DS (n = 35). Simple linear regression, Spearman r 0.3930, 95% CI: 0.05861 to 0.6482, *p< 0.05.

Starting from the inverse relationship between TGF-β1 and TNF-α plasma concentrations, the ratio between these two cytokines was then analyzed to create a composite biomarker able to characterize a model fitting with the assessment of cognitive decline. A marked reduction in the TGF-β1/TNF-α ratio was observed in young adult individuals with DS compared to age- and sex-matched HC (Figure 6A, *p* < 0.001), and a diminished TGF-β1/TNF-α ratio was evident in older adult individuals with DS, with and without AD, when compared to HC in the 35–60 years age group (Figure 6B, *p* < 0.01 for older adult DS (35–60 years) vs. HC, *p* < 0.05 for DS-AD (35–60 years) vs. HC). In addition, we found that lower TGF-β1/TNF-α ratio were strongly correlated with the rate of cognitive decline at the follow-up period (12 months) assessed by the dTSI (Figure 6C, Spearman r 0.3930, *p* < 0.05), supporting the idea proposed about the characterization of a model fitting with the assessment of cognitive decline.

### 3.4 Therapeutic concentrations of FLX rescue TGF-β1 concentrations in PBMCs from DS subjects

**FIGURE 7:**
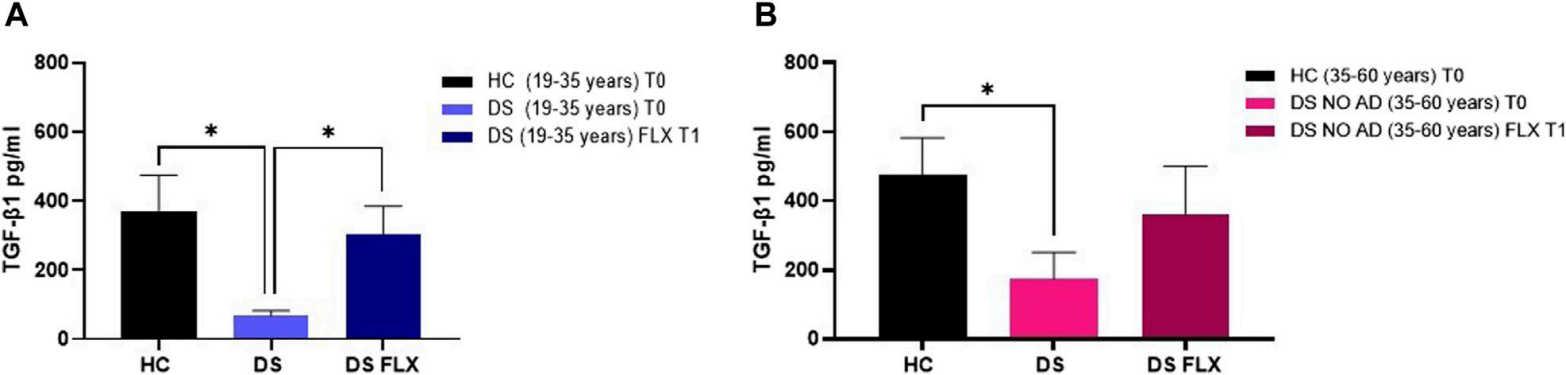
Fluoxetine was able to rescue TGF-β1 concentrations in PBMCs from individuals with DS. TGF-β1 quantification in PBMC cultured media before (T0) and after FLX treatment (T1) in **(A)** young adult individuals with DS (19–35 years) and HC (19–35 years) and **(B)** older adult individuals with DS without Alzheimer’s disease (AD)-related cognitive decline (35–60 years) and HC (35–60 years). One-way ANOVA, non-parametric test, Kruskal–Wallis test, Dunn’s *post hoc* test, *p < 0.05 DS (19–35 years) T0 vs. HC (19–35 years) T0, *p<0.05 DS (19–35 years) FLX T1 vs. DS (19–35 years) T0, *p<0.05 DS NO AD (35–60 years) T0 vs. HC (35–60 years) T0, n.s. DS NO AD (35–60 years) FLX T1 vs. DS NO AD (35–60 years) T0. All data are shown as mean ± SEM. HC (19–35 years) T0 n = 9, young adult DS (19–35 years) T0 n = 8, young adult DS (19–35 years) FLX T1 n = 8, HC (35–60 years) T0 n = 7, older adult DS (35–60 years) T0 n = 5, and older adult DS (35–60 years) FLX T1 n = 5.

FLX is an SSRI known to increase TGF-β1 concentrations in rodents (Torrisi et al., 2019), but no studies have been conducted in human cells to examine whether this drug can rescue TGF-β1 concentrations in the culture media of PBMCs from individuals with DS. In our *ex vivo* approach, PBMCs isolated from individuals with DS were treated with therapeutic concentrations of FLX at 1 µM. As a control group, PBMCs from HC subjects were cultured without FLX treatment. Notably, at baseline (T0: before FLX treatment), a significant reduction in TGF-β1 concentrations was observed in the PBMC culture media from young adult individuals with DS (19–35 years) compared to PBMCs from age- and sex-matched HC at T0 (Figure 7A, *p* < 0.05). Additionally, a deficit in TGF-β1 concentrations was found in the PBMC cultured media obtained from older adult individuals with DS (35–60 years) without AD-related cognitive decline (Figure 7B, *p* < 0.05). Interestingly, a 24-h treatment with FLX was able to rescue TGF-β1 concentrations in the culture media of PBMCs from young adult individuals with DS (19–35 years) to values comparable to the matched HC controls (Figure 7A, *p* < 0.05). However, only a trend, without statistical significance, was observed in PBMCs from older individuals with DS.

## 4 Discussion

Aging individuals with DS possess an increased risk of developing AD among other factors to the increased Aβ concentrations, resulting from the APP overexpression linked to trisomy 21. However, evidence suggests that Aβ oligomers are necessary but insufficient cause of AD, with additional cofactors, such as neuroinflammation, being associated with the onset of cognitive decline in individuals with DS (Giuffrida et al., 2010; Fessel, 2018). Indeed, several factors, such as genetic predisposition and environmental and behavioral factors, contribute to the onset of the chronic inflammatory state typical of individuals with DS (Lorenzon et al., 2023). Neuropathological *postmortem* similarities are evident in the brains of individuals with DS and AD patients. Aβ deposition, a hallmark of AD, initiates early in DS and escalates exponentially after the age of 40. Previous studies suggested that not all DS individuals manifest AD symptoms in adulthood (Iulita et al., 2016a; Salehi et al., 2016), but recent studies found that all individuals with DS will develop AD in their adulthood (average 53.8 years) and also that this is independent that there is an asymptomatic period like for neurotypical developing people, although AD progression is much faster (Iulita et al., 2022).

The diagnosis of AD-related cognitive decline in people with DS is complicated because specific tools are needed to distinguish the pre-existing intellectual disability from cognitive decline (Moran et al., 2013; Gensous et al., 2020). In this context, biomarkers may play a crucial role in differentiating AD pathophysiology in DS individuals and predicting the onset of neurodegenerative cognitive decline (Snyder et al., 2020). In recent years, efforts have been made to identify biomarkers for AD in DS individuals. Despite some progress in brain imaging and CSF-based biomarkers (Snyder et al., 2020), specific diagnostic or prognostic AD biomarkers for DS are not yet available (Lorenzon et al., 2023). Although CSF is considered a precise representation of brain changes, blood-based biomarkers offer a more accessible and less invasive method for detecting peripheral alterations early in AD (Petersen and O'Bryant, 2019; Morsiani et al., 2022). Along this line, it has been recently demonstrated by Pentz et al. that alterations in the brain nerve growth factor (NGF) metabolism are strictly reflected in both the plasma and CSF from individuals with DS (Carmona-Iragui et al., 2021; Pentz et al., 2021).

Previous studies have identified elevated plasma pro-inflammatory markers in individuals with DS years before the onset of dementia. According to this scenario, Iulita and colleagues found higher plasma ProNGF, MMP-1, MMP-3, MMP-9, IFN-γ, TNF-α, IL-6, IL-8, and IL-10 levels among individuals with DS and AD dementia as compared to healthy controls, and interestingly, plasma pro-inflammatory markers were identified as being elevated in DS individuals before the onset of dementia (Iulita et al., 2016b).

When considering the complex pathophysiology of cognitive decline in DS, we cannot ignore the eventual contribution of neurotrophic factors. In the last few years, it has been proposed that a deficit of neurotrophic factors such as NGF and TGF-β1 can contribute to the pathogenesis of AD-related cognitive decline, and a strong neurobiological link exists in the AD brain between an early pro-inflammatory process and a deficit of TGF-β1 (Iulita et al., 2016b; Caraci et al., 2017). An increasing amount of evidence suggests a neuroprotective activity for TGF-β1 against Aβ neurotoxicity (Caraci et al., 2008; Dobolyi et al., 2012; Caraci et al., 2016; Torrisi et al., 2019), but the role of TGF-β1 in cognitive decline in DS subjects has not been investigated. In this study, we hypothesized that a deficit of TGF-β1 could work as a new blood-based biomarker, predicting cognitive decline in DS individuals. Our prospective observational study revealed lower TGF-β1 plasma concentrations at baseline in both young and older adult individuals with DS without AD. Interestingly, TGF-β1 concentrations were significantly reduced in young adult individuals with DS compared to age- and sex-matched healthy controls, suggesting that a deficit of TGF-β1 might be a potential early and prolonged event in the cognitive decline pathophysiology of DS.

Regarding gender differences in DS, our findings showed a significant reduction in TGF-β1 plasma concentrations in young adult female individuals with DS compared to healthy controls, with no statistically significant deficit in young adult male individuals with DS. In older adult male individuals with DS without AD-related cognitive decline, we observed a significant reduction in TGF-β1 plasma concentrations. These observed differences could be partially due to reduced ovarian reserve associated with absence or retardation of follicle growth and/or lower concentrations of primordial cell maturation in DS (Hojager et al., 1978; Parizot et al., 2019), although in our cohort, female DS individuals present a regular menstrual cycle. We cannot exclude a high variability in our sample of adult DS subjects. It is relevant to underline that we found a significant reduction in TGF-β1 plasma concentrations in older adult male individuals with DS (35–60 years) without AD-related cognitive decline. We, therefore, believe that further studies are needed in a larger sample of individuals with DS to understand the impact of gender on TGF-β1 plasma concentrations.

The combination of biological and neuropsychological markers might be essential to identify DS individuals at a high risk of cognitive decline development as well as to establish which subjects should be included in secondary prevention studies for the assessment of future disease-modifying drugs (Iulita et al., 2016b; Hampel et al., 2018). Therefore, we examined the association between the plasma concentrations of TGF-β1 at baseline (T0) to the rate of cognitive decline (T1), and we found that the lower plasma TGF-β1 concentrations at T0 strongly predicted the following cognitive decline evaluated by the TSI score at 12 months in individuals with DS, suggesting that TGF-β1 should be further explored as a potential plasma marker able to predict the following cognitive decline in the DS population.

It is well-known that an increase in pro-inflammatory markers such as TNF-α is involved in the cognitive decline process of individuals with DS (Iulita et al., 2016b). As expected, we found that TNF-α plasma concentrations were increased in older adult DS individuals with AD-related cognitive decline compared to HCs, but more interestingly, the concentrations of TNF-α were highly elevated in young adult male and female individuals with DS about four-fold increase compared to age- and sex-matched HC.

Increased peripheral biomarkers related to inflammation along with a decrease in the anti-inflammatory cytokine concentrations have been found in the serum from individuals with DS without AD, suggesting that signatures of peripheral inflammation can be detected in an early preclinical stage of cognitive decline in DS population (Rodrigues et al., 2014). Interestingly, we report, for the first time, a negative correlation between a deficit of TGF-β1 concentrations and higher TNF-α plasma concentrations only in young adult individuals with DS. Our evidence that the TGF-β1/TNF-α ratio, considered a single composite biomarker, is reduced both in young adult individuals with DS and in older adult individuals with DS with or without AD compared to HC and that lower TGF-β1/TNF-α ratio are strongly correlated with the following cognitive decline assessed by the TSI score at T1 should be confirmed in larger observational long-term studies.

To validate TGF-β1 as a potential pharmacological target, we explored its deficit in PBMCs as an *ex vivo* surrogate model for identifying pharmacological targets in AD-related cognitive dysfunction (Rodrigues et al., 2014; Esteras et al., 2016). Interestingly, we found, for the first time, a significant deficit of TGF-β1 in PBMCs culture media from young and older adult individuals with DS. Starting from this new evidence, we then examined the hypothesis that drugs that are able to rescue TGF-β1 in rodents can restore TGF-β1 concentrations also in humans. We have previously demonstrated that fluoxetine is neuroprotective against Aβ-induced neurodegeneration via a paracrine signaling TGF-β1-mediated mechanism (Caraci et al., 2016) and is able to completely reverse depressive-like phenotype and memory deficits in Aβ-injected mice by a rescue of hippocampal TGF-β1 concentrations (Torrisi et al., 2019).

Different studies demonstrated that a chronic treatment with fluoxetine in adult models of DS increases hippocampal neurogenesis, promotes synaptic plasticity, and rescues memory impairment (Clark et al., 2006; Bianchi et al., 2010; Guidi et al., 2013; Begenisic et al., 2014). Furthermore, a neonatal treatment with fluoxetine has long-term enduring effects in Ts65Dn mice, restoring cognitive impairment and preventing early symptoms of AD-like pathology (Stagni et al., 2015), but it remains presently unknown whether chronic treatment with fluoxetine can prevent the cognitive impairment in individuals with DS by the rescue of TGF-β1 concentrations. The first step to validate the deficit of TGF-β1 in DS in our study has been to employing an *ex vivo* approach, and we found that TGF-β1 concentrations are reduced in parallel both in the plasma and in the PBMCs of individuals with DS. Interestingly, therapeutic concentrations of fluoxetine applied to cultured PBMCs (1 µM for 24 h) were able to rescue TGF-β1 concentrations in the culture media of young adult individual DS PBMCs to control PBMC values, suggesting that fluoxetine, an SSRI endowed with neuroprotective activity, might rescue TGF-β1 concentrations in adult individuals with DS at higher risk to develop AD. An open question remains to understand the impact of a chronic treatment with fluoxetine on cognitive decline in individuals with DS, also considering the high prevalence of depression in DS (Walker et al., 2011) and the role of depression as a risk factor for AD in the DS population. The limitations to our study are due to the small size of our DS cohort, which precluded performing a cross-validation analysis of the proposed blood-based biomarkers at the present time. Therefore, we cannot be certain that our proposed biomarker could act to predict cognitive decline associated with AD in a larger DS cohort. Furthermore, given that TSI was used as a global measure of cognition, it would be important to analyze in future studies the relationship between TGF-β1 and decline in specific cognitive domains, such as verbal memory and explicit long-term memory (Lott and Dierssen, 2010). Nevertheless, in the present study, we found for the first time, that both young and older adult individuals with DS exhibit lower TGF-β1 plasma concentrations at baseline combined with higher TNF-α levels. In conclusion, we believe that our data open the path to long-term observational studies in individuals with DS to validate the hypothesis that a deficit of TGF-β1 can represent a new biological marker useful to predict AD-related cognitive decline in individuals with DS and monitor the progression and/or development of cognitive decline in its prodromal phase. Most importantly, it would be relevant to assess whether a long-term treatment with appropriated doses of fluoxetine (20–40 mg/die) can improve cognitive function in DS individuals by the rescue of TGF-β1.

## Data Availability

The raw data supporting the conclusions of this article will be made available by the authors, without undue reservation.
